# Direct Immunofluorescence in Immunobullous Disorders of Skin With Histopathological Correlation Among Patients Attending a Tertiary Care Center

**DOI:** 10.7759/cureus.96265

**Published:** 2025-11-06

**Authors:** P Meilung Phom, Anbumozhi MK, Preethi M

**Affiliations:** 1 Department of Pathology, Sree Balaji Medical College And Hospital, Chennai, IND; 2 Department of Pathology, Sree Balaji Medical College and Hospital, Chennai, IND; 3 Department of Pathology, Sree Balaji Medical College, Chennai, IND

**Keywords:** autoimmune blistering diseases, bullous pemphigoid (bp), direct immunofluorescence, pemphigus foliaceous, pemphigus vulgaris

## Abstract

Background: Autoimmune blistering diseases (AIBDs) are a heterogeneous group of disorders characterized by autoantibody-mediated damage to skin and mucous membranes. Accurate diagnosis relies on a combination of clinical, histopathological, and immunopathological findings. Direct immunofluorescence (DIF) is a cornerstone in the diagnosis of AIBDs, as it enables visualization of in vivo-bound immunoreactants.

Aim: This study aimed to assess the histopathological patterns and DIF findings among patients presenting with vesiculobullous lesions, focusing on the distribution, pattern, intensity, and type of antibody deposition.

Methods: A total of 30 biopsy specimens from patients with clinically suspected vesiculobullous disorders were analyzed. Routine histopathology was performed, followed by DIF using anti-IgG, IgA, and C3 conjugates. Data on the pattern (linear basement membrane zone (BMZ) vs. intercellular (ICS)), intensity (weak, moderate, strong), and type of immunoglobulin deposition were recorded and analyzed.

Results: Among the 30 cases, bullous pemphigoid (n=13) was the most common, followed by pemphigus vulgaris (n=5) and pemphigus foliaceous (n=4). DIF showed positivity in 20 cases (66.7%). Bullous pemphigoid exhibited a linear BMZ pattern with IgG and C3 deposition, showing moderate intensity in 10 and weak intensity in 3 cases. Pemphigus vulgaris and pemphigus foliaceous showed ICS IgG deposition, with pemphigus foliaceous also demonstrating weak IgA positivity. Granular C3 dermatosis showed BMZ C3 deposition with weak intensity. A highly significant association between IgG (p = 0.0001) and C3 (p = 0.002) positivity with specific histopathological diagnoses, while IgA showed no significant association (p = 0.987).

Conclusion: DIF is an invaluable diagnostic tool that complements histopathology, enabling accurate differentiation of AIBDs based on antibody type and distribution pattern. Other blistering conditions, including subcorneal pustules and acute inflammatory lesions, showed no or minimal immunoglobulin deposition. These findings highlight the diagnostic utility of DIF in differentiating AIBDs based on the type and intensity of antibody deposition.

## Introduction

Immunobullous disorders of the skin are a heterogeneous group of autoimmune blistering diseases (AIBDs) characterized by the presence of autoantibodies directed against specific structural components of the epidermis or the dermoepidermal junction. They represent a clinically significant subset of dermatological conditions due to their chronicity, relapsing course, and potential morbidity. In India, their prevalence has been estimated to be approximately 3.2 per 100,000 population, although regional variations have been reported and the true burden is likely underestimated due to underreporting and diagnostic challenges [1].

Bullous disorders can be broadly classified into autoimmune and non-autoimmune types based on their pathogenesis [2]. Autoimmune bullous disorders (AIBDs) include pemphigus group disorders (such as pemphigus vulgaris, pemphigus foliaceus, and paraneoplastic pemphigus) and subepidermal bullous disorders (such as bullous pemphigoid, mucous membrane pemphigoid, epidermolysis bullosa acquisita, and linear IgA bullous dermatosis) [3,4]. In these conditions, autoantibodies are formed against desmosomal or hemidesmosomal proteins, disrupting cell adhesion and causing intraepidermal or subepidermal blister formation. In contrast, non-autoimmune bullous disorders (such as inherited epidermolysis bullosa) primarily arise from genetic defects in structural proteins and are usually evident from early childhood [5].

Among the AIBDs, pemphigus vulgaris and bullous pemphigoid are the two most frequently encountered disorders globally [6]. Pemphigus vulgaris is caused by IgG autoantibodies against desmoglein 3 (and occasionally desmoglein 1), leading to suprabasal acantholysis, while bullous pemphigoid involves autoantibodies against hemidesmosomal proteins BP180 and BP230, resulting in subepidermal blistering [7]. Clinically, these disorders present as fragile vesicles, bullae, and erosions over the skin and mucous membranes, often associated with pain and risk of secondary infection.

Direct immunofluorescence (DIF) is considered the gold standard diagnostic technique in AIBDs [4,8]. DIF involves applying fluorescein-labeled antibodies to frozen sections of perilesional skin biopsies to detect in situ deposits of immunoglobulins (IgG, IgA, IgM) and complement components (C3) within the epidermis or along the BMZ. The pattern of staining - such as intercellular “fish-net” fluorescence in pemphigus or linear BMZ staining in bullous pemphigoid - provides crucial clues to diagnosis [8,9]. Histopathology complements DIF by demonstrating the level and nature of blister formation and inflammatory infiltrates, but it may be inconclusive in early or partially treated cases [10]. Therefore, integrating clinical features, histopathology, and DIF findings is essential for accurate diagnosis and classification of these disorders [4].

Despite advances in diagnostic dermatopathology, considerable diagnostic overlap persists among various immunobullous disorders, as clinicopathological features often mimic one another. While histopathology remains a cornerstone in diagnosis, its findings may not always be definitive or concordant with clinical impressions, particularly in early or evolving lesions. DIF, regarded as the gold standard for diagnosing AIBDs, plays a crucial role in identifying the specific immunoreactants and deposition patterns that distinguish these entities [9,10].

However, the real-world concordance between histopathological and DIF findings can vary across populations, laboratory settings, and disease stages. In India, most existing studies on immunobullous disorders are either limited by small sample sizes, single-disease focus, or lack detailed correlation between histopathological patterns and DIF staining characteristics. Given the variability in disease presentation and resource availability across tertiary care centers, there remains a pressing need to assess how well histopathological diagnoses align with DIF outcomes in routine clinical practice.

The present study was therefore undertaken to systematically analyze the histopathological features of immunobullous skin lesions, interpret the corresponding DIF staining patterns, and evaluate the degree of correlation between the two diagnostic modalities in a tertiary care setting. This investigation seeks to provide region-specific evidence on the diagnostic value and complementarity of histopathology and DIF, thereby strengthening diagnostic accuracy and guiding optimal patient management in immunobullous disorders.

## Materials and methods

Study setting

This study was conducted in the Central Laboratory, Department of Pathology, at Sree Balaji Medical College and Hospital (SBMCH), Chennai, Tamil Nadu, India. The study included patients referred from the Department of Dermatology, SBMCH, who presented with suspected immunobullous lesions of the skin and who underwent both histopathological and DIF evaluation as part of their diagnostic workup.

Study design and duration

A cross-sectional study design was employed. The study was carried out over a period of 23 months, from August 2023 to June 2025. During this time, all eligible patients meeting the inclusion criteria were recruited, and their tissue samples were subjected to both histopathological examination and DIF analysis.

Sampling method and sample size

Purposive sampling was adopted, as only patients presenting with bullous lesions suspected to be of immunobullous etiology and who underwent both histopathology and DIF were considered for inclusion. The sample size was calculated based on the formula for the estimation of the mean score with the variance effect, where the variance (σ²) was taken as 10.5 and a 95% confidence level. Applying the formula, the final calculated sample size came to 30 patients. The sample size calculation and data analysis were carried out using OpenEpi software.

Inclusion and exclusion criteria

Patients aged above 18 years, irrespective of sex, attending the dermatology outpatient department with clinically suspected bullous lesions were included. Patients with a prior diagnosis of immunobullous disorders who were undergoing further evaluation were also included, provided they had no known history of skin or drug allergy. Patients younger than 18 years were excluded from the study. Lesions that were post-traumatic, or those occurring as a sequelae of radiotherapy or chemotherapy, were also excluded to avoid confounding pathological changes unrelated to autoimmune bullous disease.

Study tools and specimen collection

Punch biopsy samples were used as the primary study material. Fresh biopsy specimens were obtained from perilesional areas containing intact blisters to maximize diagnostic yield. In cases where fresh biopsies could not be obtained, well-preserved archival biopsy specimens fulfilling the inclusion criteria were used, provided both histopathology and DIF had been performed.

From each participant, two punch biopsies measuring at least 0.3 cm in diameter were taken under local anesthesia after aseptic preparation. Both specimens were carefully labeled with the patient’s identifying details and the site of biopsy before being transported to the central laboratory. One of the two specimens was fixed in 10% neutral buffered formalin (HiMedia Laboratories Pvt. Ltd., Mumbai, India) for routine histopathological processing, and the other was placed immediately in Michel’s medium (prepared in-house according to standard composition) for transport and preservation for DIF studies.

Histopathological Processing

Formalin-fixed tissue specimens were processed using an automated tissue processor (Leica TP1020, Leica Biosystems, Nussloch, Germany). Following fixation, tissues were dehydrated through ascending grades of alcohol, cleared in xylene (Merck Life Science Pvt. Ltd., Mumbai, India), and embedded in paraffin wax. Paraffin blocks were sectioned at 4-5 μm thickness using a rotary microtome (Leica RM2235, Leica Biosystems, Nussloch, Germany) fitted with high-profile microtome blades (Feather, Osaka, Japan). Sections were mounted on standard glass slides (75 mm × 25 mm × 1.35 mm) and dried on a slide warmer (Thermo Fisher Scientific, Waltham, MA, USA). All histopathological procedures were performed following standard hematoxylin and eosin staining protocols as described in established histopathology manuals [11]. No specialized or modified techniques were employed unless specified for individual cases.

Routine hematoxylin and eosin (H&E) staining was performed. Sections were deparaffinized in two changes of xylene (3 minutes each), rehydrated through descending grades of alcohol, and washed in running water. They were stained with Harris hematoxylin (HiMedia Laboratories Pvt. Ltd., Mumbai, India) for 5-10 minutes, differentiated in 1% acid alcohol, blued in lithium carbonate, and counterstained with 1% eosin Y (HiMedia Laboratories Pvt. Ltd., Mumbai, India) for 30 seconds. After dehydration and clearing, slides were mounted using Dibutyl Phthalate Polystyrene Xylene (DPX) mountant (Merck Life Science Pvt. Ltd., Mumbai, India) and covered with a glass coverslip. Stained slides were examined under a standard light microscope (Leica DM500, Leica Microsystems, Wetzlar, Germany) to document the level of blister formation, type of inflammatory infiltrate, and presence of acantholytic changes [12].

Direct Immunofluorescence Procedure

Specimens for DIF were transported in Michel’s transport medium, prepared according to standard composition: Solution A (1 M potassium/sodium citrate buffer, 0.1 M magnesium sulfate, 0.1 M ethylmaleimide, distilled water, and ammonium sulfate; pH adjusted to 7.2).

On receipt in the laboratory, the tissue specimens were embedded in Optimal Cutting Temperature (OCT) compound (Tissue-Tek®, Sakura Finetek Europe B.V., Alphen aan den Rijn, The Netherlands) and snap-frozen in a cryostat (Leica CM1860, Leica Biosystems, Nussloch, Germany) at −18°C. Cryostat sections of 4 μm thickness were cut and mounted on clean glass slides, with at least two sections on each slide and three slides prepared per biopsy. The slides were air-dried under an electric fan for 10 minutes and then washed gently with phosphate-buffered saline (PBS, pH 7.1-7.2; HiMedia Laboratories Pvt. Ltd., Mumbai, India).

After air drying again, the sections were incubated in a moist chamber at 37°C for 30 minutes with fluorescein isothiocyanate (FITC)-conjugated antibodies directed against human immunoglobulins and complement components. Specifically, the following FITC-labeled antisera were used: anti-IgG (Dako, Agilent Technologies, Santa Clara, CA, USA), anti-IgA (Sigma-Aldrich, St. Louis, MO, USA), and anti-C3 (Thermo Fisher Scientific, Waltham, MA, USA), diluted in PBS. After incubation, the sections were washed thrice in PBS to remove unbound antibody, dried, and mounted in buffered glycerol (Sigma-Aldrich, St. Louis, MO, USA).

The slides were examined using a fluorescence microscope (Leica DM1000, Leica Microsystems, Wetzlar, Germany)equipped with an HG 50 UV lamp (Leica Microsystems) and a blue excitation filter. FITC emitted characteristic apple-green fluorescence when excited by UV light. The presence, pattern (linear, granular, or intercellular), and intensity of fluorescence were recorded, with positivity graded subjectively as strong (+++), moderate (++), or weak (+). After sectioning, the remaining OCT-embedded tissue was wrapped in aluminum foil and stored at −20°C until the end of the study period for potential repeat or confirmatory testing.

Ethical considerations

The study was initiated after obtaining clearance from the Institutional Ethics Committee of SBMCH, Chennai (002/SBMC/IHEC/2023/1998). Written informed consent was obtained from all participants prior to enrollment after explaining the nature, purpose, and possible risks of the study. Confidentiality and anonymity of patient data were maintained throughout, and all procedures were conducted in accordance with the ethical principles of the Declaration of Helsinki.

Statistical analysis

All clinical, histopathological, and DIF data were entered into a spreadsheet and analyzed using SPSS ver 26.0. Descriptive statistics were used to summarize demographic and clinical data. To find the significance in qualitative categorical data, the Chi-Square/Fisher’s exact test was used. In the above statistical tool, a p-value <0.05 was considered statistically significant.

## Results

In the present study, a total of 30 patients with suspected immunobullous disorders of the skin (13 cases were bullous pemphigoid, 5 cases were pemphigus vulgaris, 4 cases were pemphigus foliaceous, 2 cases were acute exanthematous pustulosis, 2 cases were subcorneal pustules, 2 cases were blister with acute inflammatory cells, 1 case was Granular C3 dermatosis and 1 case was intraepithelial bullous lesion) were enrolled between August 2023 and May 2025, and their biopsy samples were subjected to both histopathological examination and DIF testing. The age of the study participants ranged from below 40 years to above 80 years. The majority of the cases were observed in the age group of 61-70 years, comprising eight patients (26.7%) and two patients (6.7%) above 80 years, being the lowest. With respect to gender distribution, females constituted a slightly higher proportion with 17 cases (56.7%), while males accounted for 13 cases (43.3%) (Table 1).

**Table 1:** Age and gender distribution of study participants (n=30)

Variable	Category	Frequency (n)	Percentage (%)
Age (years)	< 40	3	10.0
	41–50	7	23.3
	51–60	6	20.0
	61–70	8	26.7
	71–80	4	13.3
	> 80	2	6.7
Gender	Female	17	56.7
	Male	13	43.3

Among the 30 cases evaluated histopathologically, bullous pemphigoid was the most frequent diagnosis (43.3%), all of which were DIF-positive. Pemphigus group disorders accounted for 30% of cases, with DIF positivity seen in 60% of pemphigus vulgaris and 75% of pemphigus foliaceous cases. Other histologies, including acute exanthematous pustulosis, subcorneal pustules, and blister with acute inflammatory cells (each 6.7%), were all DIF-negative. A single case each of granular C3 dermatosis (positive) and intraepithelial bullous lesion (negative) was noted. Overall, DIF positivity was observed in 66.7% (20/30) of cases, while 33.3% (10/30) were DIF-negative (Table 2).

**Table 2:** Distribution of histopathological diagnoses and their direct immunofluorescence (DIF) positivity status

Histopathology Diagnosis	Total n (%)	DIF Positive n (%)	DIF Negative n (%)
Bullous Pemphigoid	13 (43.3%)	13 (100.0%)	0 (0.0%)
Pemphigus Vulgaris	5 (16.7%)	3 (60.0%)	2 (40.0%)
Pemphigus Foliaceous	4 (13.3%)	3 (75.0%)	1 (25.0%)
Acute Exanthematous Pustulosis	2 (6.7%)	0 (0.0%)	2 (100.0%)
Subcorneal Pustules	2 (6.7%)	0 (0.0%)	2 (100.0%)
Blister With Acute Inflammatory Cells	2 (6.7%)	0 (0.0%)	2 (100.0%)
Granular C3 Dermatosis	1 (3.3%)	1 (100.0%)	0 (0.0%)
Intraepithelial Bullous Lesion	1 (3.3%)	0 (0.0%)	1 (100.0%)
Total	30 (100%)	20 (66.7%)	10 (33.3%)

In Table 3, the pattern and staining intensity of DIF expression varied across the different immunobullous disorders. All bullous pemphigoid cases demonstrated a linear basement membrane zone (BMZ) pattern, with moderate staining intensity observed in 10 cases and weak intensity in 3 cases. Pemphigus vulgaris (PV) exhibited an intercellular (ICS) pattern in three cases, all showing moderate staining intensity. Pemphigus foliaceous (PF) also showed an ICS pattern, with two cases displaying moderate and one case showing weak staining intensity. Additionally, the single case of granular C3 dermatosis showed a BMZ pattern with weak staining intensity.

**Table 3:** Direct immunofluorescence (DIF) findings among various immunobullous disorders of skin BMZ: Basement Membrane Zone; ICS: intercellular substance

Disorder	No of cases	Pattern	Intensity of staining		Antibodies detected			
			Mod strong	Weak	IgG	IgA	C3	IgG+C3
Bullous Pemphigoid	13	Linear BMZ	10	3	0	0	0	13
Pemphigus Vulgaris	5	ICS	3	0	0	1	0	2
Pemphigus Foliaceous	4	ICS	2	1	2	1	0	0
Acute Exanthematous Pustulosis	2	-	0	0	0	0	0	0
Subcorneal Pustules	2	-	0	0	0	0	0	0
Blister With Acute Inflammatory Cells	2	-	0	0	0	0	0	0
Granular C3 Dermatosis	1	BMZ	1	0	0	0	1	0
Intraepithelial Bullous Lesion	1	-	0	0	0	0	0	0

**Figure 1:**
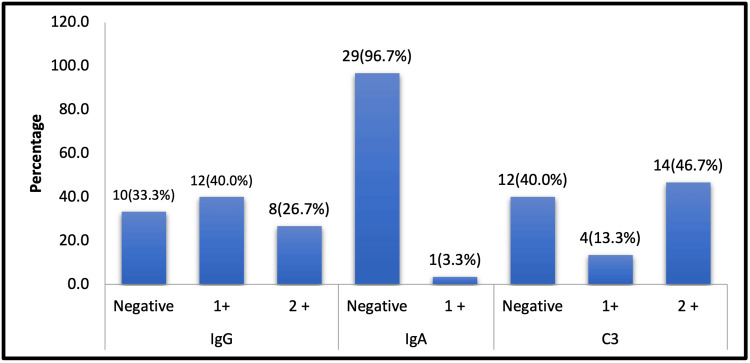
Comparison of immunobullous grading The image is represented in n(%)

In the present study, the distribution of immunobullous grading findings was as follows: IgG was negative in 10 cases (33.3%), weakly positive in 12 cases (40.0%), and moderately positive in 8 cases (26.7%). IgA showed negativity in 29 cases (96.7%) and weak positivity in only 1 case (3.3%). C3 was negative in 12 cases (40.0%), weakly positive in 4 cases (13.3%), and moderately positive in 14 cases (46.7%). Among the immunoreactants, C3 demonstrated the highest rate of positivity (46.7%), whereas IgA exhibited the lowest (3.3%) (Figure 1).

Table 4 presents the distribution of immunoreactant (IgG, IgA, and C3) deposition patterns detected by DIF across various histopathological examination (HPE) diagnoses, along with their statistical significance. Among the 30 cases, IgG deposits were most frequently observed in bullous pemphigoid (12 cases with 1+ and 1 case with 2+ intensity) and pemphigus vulgaris (3 cases with 2+), while all subcorneal pustule, acute exanthematous pustulosis, and intraepithelial bullous lesion cases were IgG negative. IgA deposits were rare, detected only in a single case, and showed no statistically significant association with any HPE category (p = 0.987). C3 deposits were predominantly seen in bullous pemphigoid (12 cases with 2+), with a significant association observed between C3 positivity and HPE diagnosis (p = 0.002). Overall, there was a highly significant association between IgG positivity and specific histopathological diagnoses (χ² = 42.418, p = 0.0001), while IgA showed no significant association, and C3 showed a strong association (χ² = 33.956, p = 0.002).

**Table 4:** Correlation of histopathological diagnosis with direct Immunofluorescence findings (IgG, IgA, C3) including Chi-Square and p-values Chi-square/Fisher’s exact test #No statistical significance (p-value <0.05) *High statistical significance (p-value <0.01)

HPE Diagnosis	IgG Negative n (%)	IgG 1+ n (%)	IgG 2+ n (%)	Chi-square	p-value IgG	IgA Negative n (%)	IgA 1+ n (%)	Chi-square	p-value	C3 Negative n (%)	C3 1+ n (%)	C3 2+ n (%)	Chi-square	p-value C3
Subcorneal Pustules	2 (6.7)	0 (0.0)	0 (0.0)	42.418	0.0001**	2 (6.7)	0 (0.0)	1.353	0.987#	2 (6.7)	0 (0.0)	0 (0.0)	33.956	0.002**
Blister w/ Acute Inflammatory Cells	0 (0.0)	0 (0.0)	2 (6.7)			2 (6.7)	0 (0.0)			2 (6.7)	0 (0.0)	0 (0.0)		
Bullous Pemphigoid	0 (0.0)	12 (40.0)	1 (3.3)			12 (40.0)	1 (3.3)			0 (0.0)	1 (3.3)	12 (40.0)		
Granular C3 Dermatosis	1 (3.3)	0 (0.0)	0 (0.0)			1 (3.3)	0 (0.0)			1 (3.3)	0 (0.0)	1 (3.3)		
Pemphigus Foliaceous	1 (3.3)	0 (0.0)	3 (10.0)			4 (13.3)	0 (0.0)			4 (13.3)	0 (0.0)	0 (0.0)		
Pemphigus Vulgaris	2 (6.7)	0 (0.0)	3 (10.0)			5 (16.7)	0 (0.0)			2 (6.7)	3 (10.0)	0 (0.0)		
Skin w/ Subcorneal Pustule-Acute Exanthem	2 (6.7)	0 (0.0)	0 (0.0)			2 (6.7)	0 (0.0)			2 (6.7)	0 (0.0)	0 (0.0)		
Intraepithelial Bullous Lesion	1 (3.3)	0 (0.0)	0 (0.0)			1 (3.3)	0 (0.0)			1 (3.3)	0 (0.0)	0 (0.0)		
Total	10 (33.3)	12 (40.0)	8 (26.7)			29 (96.7)	1 (3.3)			12 (40.0)	4 (13.3)	14 (46.7)		

**Figure 2:**
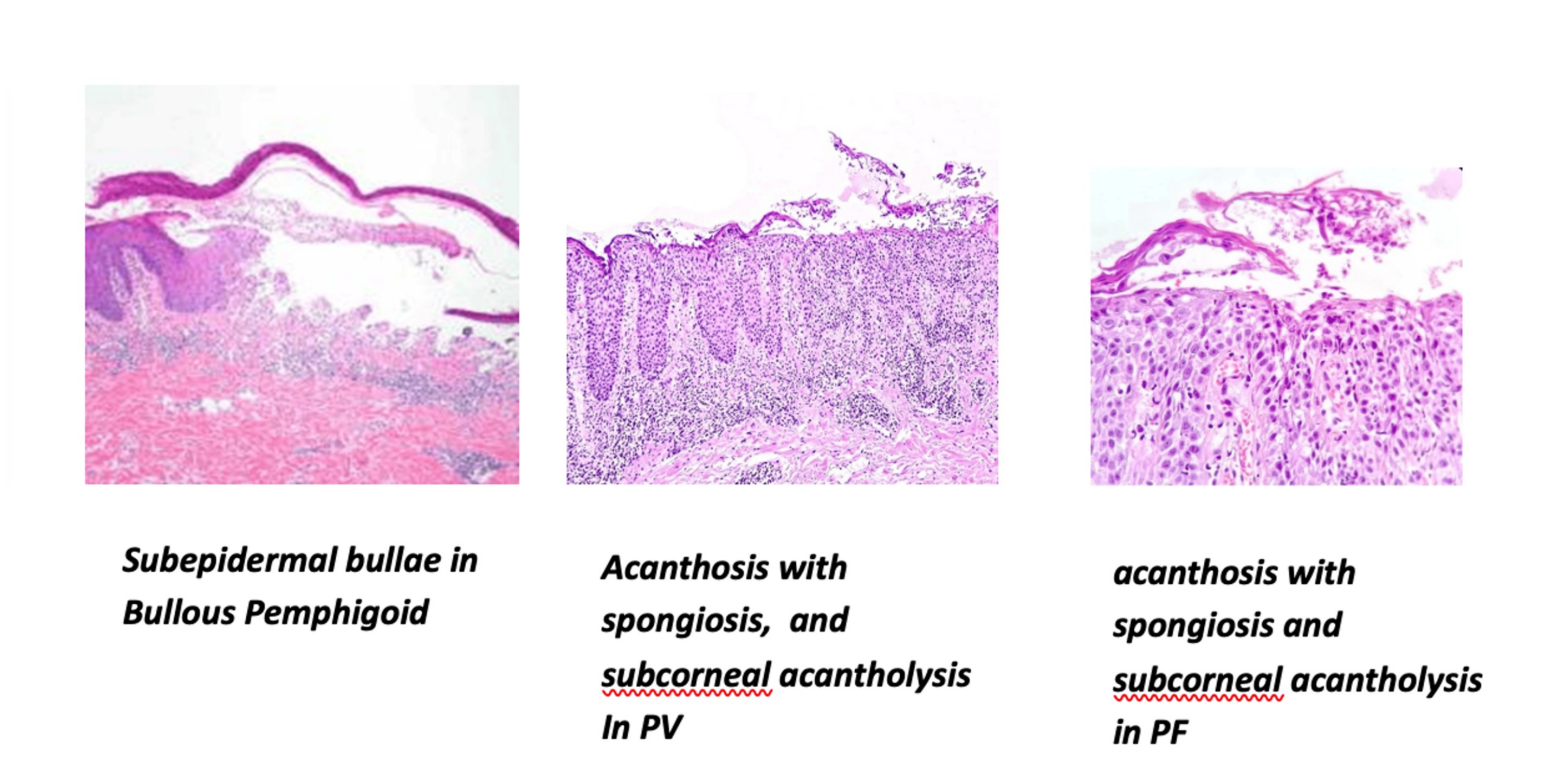
HPE changes observed in bullous pemphigoid, pemphigus vulgaris, and foliaceous PV: pemphigus vulgaris; HPE: histopathological examination

Figure 2 shows key histopathological features of major autoimmune blistering disorders. Bullous pemphigoid (BP) exhibits subepidermal bullae with dermal-epidermal separation and eosinophilic infiltrate. PV shows suprabasal acantholysis with acanthosis and spongiosis, while PF demonstrates subcorneal acantholysis, acanthosis, and spongiosis, leading to superficial intraepidermal blisters.

## Discussion

This autoimmune attack leads to the formation of cutaneous as well as mucosal blisters. These conditions are often chronic, potentially debilitating, and in severe cases, life-threatening. Hence, timely diagnosis and early therapeutic intervention are crucial for preventing complications and improving patient outcomes. DIF has emerged as a pivotal diagnostic tool for evaluating vesiculobullous lesions of the skin, supplementing clinical and histological assessment. DIF allows the in-situ demonstration of immune deposits such as immunoglobulins and complement components, helping to differentiate between the various immunobullous entities that can otherwise have overlapping histopathological features. In this study, we evaluated 30 patients who underwent both HPE and DIF analysis to establish a clinicopathological correlation and to assess the diagnostic utility of DIF in cases with inconclusive or discordant histopathological findings.

In our study cohort, BP emerged as the most common lesion, accounting for 13 out of 30 cases (43.33%), with a mean age of presentation around 60 years. This observation is consistent with previous studies and literature from Western populations, which also document BP as the most prevalent AIBD, particularly in the elderly age group [13,14]. Women were more commonly affected (69.2%), aligning with the known slight female predominance reported in other epidemiological studies. Histopathology showed subepidermal bullae with eosinophil-predominant infiltrates (seen in 8 out of 13 cases), accompanied by scattered lymphocytes within the bullous cavity. DIF demonstrated a classic linear deposition of IgG and complement component C3 along the BMZ in all 13 cases. This is in agreement with the findings of Robert [15], who described C3 deposits in nearly all cases of BP, contrasting with PV, where DIF negativity can occasionally occur. The co-deposition of IgG and C3 underscores the pathogenic role of complement activation in BP.

The second most common lesion was pemphigus vulgaris, comprising 5 cases (13.33%), with a mean age of 34 years. This aligns with the demographic pattern described in both Indian and Western literature [13,16]. Histopathology showed suprabasal clefting with scattered acantholytic keratinocytes, an inflammatory infiltrate of lymphocytes and neutrophils within the bulla, and perivascular mononuclear cell infiltrates in the dermis. DIF was positive in three cases, showing a strong intercellular staining (ICS) pattern of IgG throughout the epidermis. Interestingly, none of our cases showed a linear BMZ pattern, although rare such cases have been documented by Kumar et al [17]. Two of the positive cases showed combined IgG and C3 staining, and one case showed IgG and IgA co-deposition. The presence of isolated IgA or IgM has been reported by Venning, but was not observed in our series [18]. Arya et al. had reported that complement deposition, particularly C3, contributes to the pathogenesis of PV, which was corroborated by our finding of C3 positivity in the DIF-positive PV cases [19].

Two PV cases were negative on DIF despite showing classical histological features. Both were biopsied from perilesional skin as per standard protocol. Considering the strong clinical suspicion and HPE findings, they were managed as PV and advised for repeat DIF, but follow-up was lost. These false-negative DIF results might be attributed to sampling errors, loss of epidermis during biopsy, or early re-epithelialization at the biopsy site, which are recognized causes of DIF negativity.

Pemphigus foliaceous constituted four cases (13.33%), all between the ages of 51 and 70 years. This age and sex distribution is within the range documented in previous studies [13,20]. Histopathology in three cases showed classic subcorneal intraepidermal bullae with acantholytic keratinocytes, fibrin, and sparse inflammatory cells. DIF in these cases showed a distinct ICS staining confined to the upper two-thirds of the epidermis, typical of PF. One case showed discordant findings: histopathology showed only subcorneal neutrophilic pustules and spongiosis without acantholysis, and DIF was negative. Clinically, pustular psoriasis was considered a differential. Given negative DIF and absence of acantholysis, a diagnosis favoring pustular psoriasis was rendered. This highlights the known diagnostic pitfall where PF can sometimes show only neutrophilic collections without evident acantholysis, potentially mimicking subcorneal pustular dermatoses.

We also encountered one case of granular C3 dermatosis, a 49-year-old male who initially presented with pustular lesions progressing to erosions on the oral mucosa, followed by vegetative plaques over both axillae. Histopathology showed basket weave hyperkeratosis, acanthosis, eosinophilic microabscesses, intraepidermal spongiotic vesicles filled with neutrophils and eosinophils, and a subepidermal blister. DIF revealed a strong granular C3 deposition along the BMZ with a diffuse ICS pattern throughout the epidermis. This clinched the diagnosis, which was otherwise unclear from clinical and histological findings alone. Hashimoto et al. have emphasized the need for more clinical and experimental studies to better understand the pathogenic significance of granular C3 deposits on the BMZ [21]. This case underlines the decisive role of DIF in resolving diagnostically ambiguous cases.

Overall, 10 cases were negative on DIF despite clinical suspicion of immunobullous disease. One such case showed subepidermal bulla with eosinophils and neutrophils on HPE, raising differential diagnoses of BP and dermatitis herpetiformis. The patient was treated as BP based on histology, but refused a repeat biopsy for DIF. Another case showed only dermis on histology, lacking the epidermal component, which likely got detached during biopsy or tissue processing; the DIF was negative. The other DIF-negative cases may represent patients in early disease stages when immune deposits are scant, or those already on treatment leading to clearance of immune deposits, or they may reflect technical errors such as improper biopsy site selection or issues during cryosectioning. As recommended in the literature, repeat DIF from a new perilesional site is warranted in such cases; however, some patients in our study were lost to follow-up or declined repeat biopsies.

Our study is limited by its small sample size (n = 30), which restricts the generalizability of the findings. Follow-up data were incomplete for some patients, especially for those who were DIF-negative, which limited our ability to confirm the clinical outcomes or repeat testing. Additionally, interobserver variability in interpreting DIF staining intensity and patterns could have influenced the results. Lack of correlation with serological tests for circulating autoantibodies (like indirect immunofluorescence or ELISA) is another limitation.

Despite these limitations, our findings reinforce the critical role of DIF in the diagnosis of immunobullous disorders, especially when histopathology is inconclusive or discordant with the clinical picture. DIF not only helps distinguish between intraepidermal and subepidermal blistering diseases but also guides early and appropriate therapeutic interventions, thereby improving patient outcomes. Incorporating routine DIF testing into the diagnostic algorithm of suspected immunobullous disorders in tertiary care settings is strongly recommended.

## Conclusions

The DIF analysis revealed distinct immunoglobulin deposition patterns corresponding to specific histopathological diagnoses. BP cases predominantly exhibited strong IgG (2+) and C3 (2+) positivity, demonstrating a characteristic linear BMZ pattern. In contrast, PV and pemphigus foliaceus showed intercellular IgG deposition, with the latter also displaying occasional weak IgA deposits. Other blistering conditions, such as subcorneal pustules and acute inflammatory lesions, showed minimal or absent immunoglobulin deposition. These findings reaffirm the pivotal role of DIF as a definitive diagnostic adjunct in differentiating AIBDs, particularly in histologically ambiguous cases. Future studies integrating DIF with emerging molecular or serological assays could further refine diagnostic precision and enhance early disease stratification and management.
